# The Influence of Obesity Hypoventilation Syndrome on the Outcomes of Patients With Diabetic Ketoacidosis

**DOI:** 10.7759/cureus.25157

**Published:** 2022-05-20

**Authors:** Meghana Pattipati, Goutham Gudavalli, Lohitha Dhulipalla

**Affiliations:** 1 Internal Medicine, The Brooklyn Hospital Center, Brooklyn, USA; 2 Critical Care Medicine, Rapides Regional Medical Center, Alexandria, USA

**Keywords:** diabetic ketoacidosis, diabetes, obesity hypoventilation syndrome, osa, obesity

## Abstract

Purpose: The effect of comorbid obesity hypoventilation syndrome (OHS) on hospitalized patients with diabetic ketoacidosis (DKA) has not been studied so far. This study elucidates the outcomes of DKA patients with OHS compared to those without OHS.

Methods: Patients above 18 years of age were included in the study. The National Inpatient Sample (NIS) database of 2017 and 2018 was used and data were extracted using the International Classification of Diseases, Tenth Revision (ICD-10) codes; OHS ICD-10 code being “E66.2” and DKA ICD-10 codes being “E08.1, E09.1, E10.1, E11.1, and E13.1.” The comorbid medical conditions were also identified using the ICD-10 codes. Logistic regression analysis was performed to examine the impact of OHS on in-hospital outcomes of DKA patients.

Results: OHS was prevalent in 0.61% of the general population, as per the NIS database in the years 2017 and 2018. Primary outcomes of the study were in-hospital mortality, whereas secondary outcomes included acute kidney failure, the requirement for invasive mechanical ventilation, length of stay, and cost of hospitalization. OHS in DKA patients was associated with increased mortality (odds ratio (OR): 4.35 (2.63-7.20), p < 0.00001; adjusted OR (aOR): 1.79 (1.01-3.15), p < 0.044), acute kidney failure (OR: 2.44 (1.79-3.33), p < 0.00001; aOR: 1.43 (1.03-2.00), p < 0.031), invasive mechanical ventilation (OR: 4.17 (2.90-5.98), p < 0.00001; aOR: 1.62 (1.08-2.41), p < 0.017), increased length of stay (10.02 ± 12.42 vs. 4.70 ± 6.31, p < 0.00001), and cost of care (132314 ± 197111.8 vs. 54245.06 ± 98079.89, p < 0.00001). All-cause mortality of patients with DKA and OHS using the Cox proportional hazards ratio was 1.70 (1.02-2.84, p < 0.024) after adjusting for age, race, sex, smoking, obesity, and comorbidities such as heart failure, hypertension, chronic obstructive pulmonary disease, chronic ischemic heart disease, chronic kidney disease, liver disease, and cerebral infarction.

Conclusion: OHS is an independent risk factor for mortality in DKA, irrespective of the degree of obesity. Further prospective studies are recommended to study the effects of different treatment modalities of OHS such as identification of the need for early non-invasive ventilation or for early invasive mechanical ventilation to improve outcomes in DKA patients.

## Introduction

Obesity is a major risk factor for diabetes. Diabetic ketoacidosis (DKA) is commonly seen in patients with type 1 diabetes mellitus. According to a study in recent years, an increasing incidence of DKA has been reported without a precipitating cause, especially in people of all age groups with type 2 diabetes. These subjects are usually obese and have a strong family history of diabetes. These subjects are usually referred to as patients with Flatbush diabetes and ketosis-prone type 2 diabetes [1]. Obesity also predisposes patients to obesity hypoventilation syndrome (OHS), which is defined as obesity (BMI ≥ 30) and chronic alveolar hypoventilation leading to daytime hypercapnia and hypoxia (partial pressure of carbon dioxide ≥ 45 and partial pressure of oxygen < 70 mmHg) and sleep-disordered breathing in the absence of significant lung or respiratory muscle disease [2]. The morbidity and mortality are high for patients with OHS [2]. Patients with OHS are more likely to be diagnosed with congestive heart failure, angina pectoris, and cor pulmonale and are at a higher risk for admission to ICU and invasive mechanical ventilation than the population with a similar degree of obesity without hypoventilation. Prevalence of obesity is increasing overall and is 20% in ICU patients [3]. Obesity is associated with an increased risk of acute kidney injury (AKI) [3]. The impact of obesity on ICU mortality is debated [3]. DKA is an acute life-threatening complication of diabetes, leading to severe electrolyte abnormalities and dehydration, if not corrected in a timely fashion, leading to adverse outcomes. The aim of this study is to improve our understanding of the influence of OHS on the outcomes in patients admitted with DKA. There are no prior studies done on patients with OHS and DKA.

## Materials and methods

Data source

We utilized the Agency for Healthcare Research and Quality's (AHRQ) National Inpatient Sample (NIS) database, which is developed as part of the Healthcare Cost and Utilization Project (HCUP). NIS is the largest all-payer inpatient healthcare database in the United States. It includes data from approximately 7 million patient hospital stays per year from over 1,000 hospitals and is a representative sample of about 20% of non-federal hospitals in the United States.

Patient population

The aim of this study is to improve our understanding of the factors that influence the outcomes of DKA patients with underlying OHS in comparison with DKA patients without OHS. This is a retrospective cohort study of the NIS database, which includes the years 2017 and 2018. The International Classification of Diseases, Tenth Revision, Clinical Modification (ICD-10-CM) for both DKA and OHS is used. All patients above the age of 18 years with the diagnosis of DKA were extracted using the International Classification of Diseases, Tenth Revision (ICD-10) codes “E08.1, E09.1, E10.1, E11.1, and E13.1” and included in the study. Patients with OHS are identified using the ICD-10 diagnostic code “E66.2.” Baseline demographics and social variables such as age, gender, race, smoking history, various comorbidities (hypertension, obesity, liver disease, chronic lung disease, chronic kidney disease (CKD), cerebral infarction, heart failure, atrial fibrillation and atrial flutter, chronic ischemic heart disease, chronic obstructive pulmonary disease (COPD), obstructive sleep apnea (OSA), and BMI), and social factors like insurance payer, hospital bed size, socioeconomic status based on household income, location, region of the hospital, and teaching status were included in the analysis.

Statistical analysis

The primary outcome of the study is to estimate in-hospital mortality. Secondary outcomes of interest are acute respiratory failure, acute kidney failure, length of stay, need for mechanical ventilation, and cost of care during hospitalization. Multivariate logistic regression is used to adjust for potential confounders including age, gender, race, smoking, heart failure, COPD, hypertension, obesity, atrial fibrillation and atrial flutter, CKD, chronic liver disease, cerebral infarction, ischemic heart disease, and socioeconomic status. STATA/SE version 17.0 (StataCorp LLC, College Station, TX) is used for the analysis of the data.

## Results

In the years 2017 and 2018, the total hospitalizations with a primary diagnosis of DKA were 60,607 and the concomitant diagnoses of OHS were 172. The mean age of patients without OHS was 45.4 ± 17.8 years (range: 18-90 years) and the mean age of patients with OHS was 56.25 ± 13.47 years (range: 23-90 years) (p < 0.0001). Patients with OHS are relatively older at the time of admission. Table 1 describes the baseline demographics of the patients admitted with DKA with and without OHS. Approximately 57-65% of the patients were Caucasians in both groups. In the OHS group, there were more females than those in the without OHS group (55% vs. 49.5%, p < 0.132); however, it was not statistically significant. Greater number of patients in the OHS group had comorbidities such as hypertension (84% vs. 52%, p < 0.00001), heart failure (50% vs. 9.4%, p < 0.0001), atrial fibrillation and atrial flutter (25.6% vs. 6.2%, p < 0.00001), COPD (32.6% vs. 7.4%, p < 0.00001), OSA (15.7% vs. 3.7%, p < 0.00001), CKD (38.4% vs. 17.7%, p < 0.00001), liver disease (14.5% vs. 6%, p < 0.00001), cerebral infarction (4% vs. 1.5%, p < 0.0052), chronic ischemic heart disease (22.1% vs. 13.5%, p < 0.0009), and BMI between 30 and 40 (15.12% vs. 6.2%, p < 0.00001) and BMI ≥ 40 (70.35% vs. 4.5%, p < 0.00001).

**Table 1:** Baseline demographics and comorbidities in patients admitted with diabetic ketoacidosis (DKA) (n = 60,607) with and without obesity hypoventilation syndrome (OHS) COPD, chronic obstructive pulmonary disease; CKD, chronic kidney disease; LOS, length of stay.

Variables	DKA without OHS (n = 60,435)	Percentage (%)	DKA with OHS (n = 172)	Percentage (%)	P-value
Sex					
Male	30,517	50.5%	77	44.77%	p < 0.133
Female	29,912	49.5%	95	55.23%	p < 0.132
Age group					
≥18 to <20	2,398	3.97%	0	0	-
≥20 to <30	12,556	20.78%	7	4.07%	p < 0.001
≥30 to <40	10,352	17.13%	16	9.30%	p < 0.006
≥40 to <50	10,018	16.58%	25	14.53%	p < 0.472
≥50 to <60	10,809	17.89%	46	26.74%	p < 0.002
≥60 to <70	7,929	13.12%	54	31.40%	p < 0.001
≥70 to <80	4,343	7.19%	21	12.21%	p < 0.010
≥80 to <90	1,726	2.86%	2	1.16%	p < 0.182
≥90	304	0.50%	1	0.58%	p < 0.884
Mean age	45.40 ± 17.83		56.25 ± 13.47		p < 0.001
Race					
White	33,956	57.44%	111	65.29%	p < 0.027
Black	14,395	24.35%	38	38	p < 0.596
Hispanic	7,675	12.98%	15	15	p < 0.117
Asian/Pacific Island	924	1.56%	0	0	-
Native American	608	1.03%	1.03%	2	p < 0.837
Other	1,557	2.63%	2.63%	4	p < 0.836
Pay					
Medicare	17,295	28.66%	84	49.12%	p < 0.001
Medicaid	18,556	30.75%	46	26.90%	p < 0.261
Private insurance	15,676	25.98%	30	17.54%	p < 0.011
Self-pay	6,686	11.08%	7	4.09%	p < 0.003
No charge	479	0.79%	0	0	-
Other	1,654	2.74%	4	2.34%	p < 0.741
Household income					
0 to 25th percentile	21,943	37.09%	61	36.09%	p < 0.818
26th to 50th percentile	16,777	28.36%	56	33.14%	p < 0.160
51st to 75th percentile	12,544	21.20%	33	19.53%	p < 0.612
76th to 100th percentile	7,899	13.35%	19	11.24%	p < 0.431
Smoking	15,721	26%	27	15.7%	p < 0.002
Bed size					
Small	13,527	22.38%	30	17.44%	p < 0.120
Medium	18,154	30.04%	48	29.91%	p < 0.542
Large	28,754	47.58%	94	54.65%	p < 0.063
Location of hospital/teaching status					
Rural	6,526	10.80%	11	6.40%	p < 0.063
Urban non-teaching	13,212	21.86%	34	19.77%	p < 0.507
Urban teaching	40,697	67.34%	127	73.84%	p < 0.069
Hospital region					
Northeast	8,924	14.77%	29	16.86%	p < 0.439
Midwest	13,027	21.56%	44	25.58%	p < 0.199
South	26,226	43.40%	65	37.79%	p < 0.138
West	12,258	20.28%	34	19.97%	p < 0.867
Diabetes	60,435	100%	172	100%	p < 0.001
Obesity	7,963	13.18%	172	100%	p < 0.001
Hypertension	31,486	52.10%	144	83.72%	p < 0.001
Heart failure	5,551	9.19%	86	50%	p < 0.001
Atrial fibrillation/atrial flutter	3,739	6.19%	44	25.58%	p < 0.001
COPD	4,442	7.35%	56	32.56%	p < 0.001
CKD	10,712	17.72%	66	38.37%	p < 0.001
Liver disease	3,630	6.01%	25	14.53%	p < 0.001
Cerebral infarction	897	1.48%	7	4.07%	p < 0.005
Chronic ischemic heart disease	8,127	13.45%	38	22.09%	p < 0.0009
BMI					
1 (30-40)	3,743	6.19%	26	15.12%	p < 0.001
2 (>40)	2,731	4.52%	121	70.35%	p < 0.001
Total charges	54245.06 ± 98079.89		132314 ± 197111.8		p < 0.001
LOS	4.70 ± 6.31		10.02 ± 12.42		p < 0.001

Table 2 describes the in-hospital outcomes of patients with DKA and OHS. We observed a significant increase in the in-hospital mortality (odds ratio (OR): 4.35 (2.63-7.20), p < 0.00001; adjusted OR (aOR): 1.79 (1.01-3.15), p < 0.044), acute kidney failure (OR: 2.44 (1.79-3.33), p < 0.00001; aOR: 1.43 (1.03-2.00), p < 0.031), and mechanical ventilation (OR: 4.17 (2.90-5.98), p < 0.00001; aOR: 1.62 (1.08-2.41), p < 0.017) in patients with OHS in comparison to patients without OHS. The length of stay in DKA patients with comorbid OHS was 10.02 ± 12.42, whereas in DKA patients without OHS, it was 4.70 ± 6.31 (p < 0.00001). The cost of care was 132314 ± 197111.8 in DKA patients with OHS vs. 54245.06 ± 98079.89 in DKA patients without OHS (p < 0.00001) (Table 1). Incidence of acute respiratory failure was higher in the OHS group in the unadjusted OR; however, no statistically significant difference was found after adjusting for various variables (OR: 4.089 (2.90-5.75), p < 0.00001; aOR: 1.36 (0.93-2.00), p < 0.106). Both the primary and secondary outcomes are adjusted for age, sex, race, smoking, insurance, and socioeconomic status based on household income, and various comorbidities such as hypertension, obesity, heart failure, COPD, atrial fibrillation and atrial flutter, CKD, cerebral infarction, liver disease, and chronic ischemic heart disease.

**Table 2:** In-hospital outcomes for patients admitted with diabetic ketoacidosis (n = 60,607) with and without obesity hypoventilation syndrome

Outcomes	Odds ratio	P-value	Adjusted odds ratio	P-value
In-hospital mortality	4.35 (2.63-7.20)	<0.00001	1.79 (1.01-3.15)	<0.044
Acute respiratory failure	4.089 (2.90-5.75)	<0.00001	1.36 (0.93-2.00)	<0.106
Mechanical ventilation	4.17 (2.90-5.98)	<0.00001	1.62 (1.08-2.41)	<0.017
Acute kidney failure	2.44 (1.79-3.33)	<0.00001	1.43 (1.03-2.00)	<0.031

NIS is the largest inpatient database representing >97% of the US population. Utilizing this database, we showed the following significant findings: (1) patient characteristics such as age, smoking, hypertension, heart failure, atrial fibrillation, atrial flutter, OSA, COPD, CKD, liver disease, cerebral infarction, chronic ischemic heart disease, and BMI are significantly different in OHS patients compared to non-OHS patients (p < 0.05). (2) The prevalence of OHS in DKA patients is 0.28%. (3) OHS in DKA patients was associated with increased mortality (OR: 4.35 (2.63-7.20), p < 0.00001; aOR: 1.79 (1.01-3.15), p < 0.044), acute kidney failure (OR: 2.44 (1.79-3.33), p < 0.00001; aOR: 1.43 (1.03-2.00), p < 0.031), and mechanical ventilation (OR: 4.17 (2.90-5.98), p < 0.00001; aOR: 1.62 (1.08-2.41), p < 0.017). (4) OHS in DKA patients is associated with increased length of stay (10.02 ± 12.42 vs. 4.70 ± 6.31, p < 0.00001) and cost of care (132314 ± 197111.8 vs. 54245.06 ± 98079.89, p < 0.00001). (5) All-cause mortality of patients with DKA and OHS calculated using the Cox proportional hazards ratio was 1.70 (1.02-2.84, p < 0.024) after adjusting for age, race, sex, smoking, obesity, and comorbidities such as heart failure, hypertension, COPD, chronic ischemic heart disease, CKD, liver disease, and cerebral infarction. The baseline characteristics revealed no significant difference in males and females in the two groups (the p-value was not statistically significant).

## Discussion

The effects of obesity on diabetes and metabolic syndrome are well known. The prevalence of OHS in the general population according to the NIS database in 2017 and 2018 was 0.61%. A condition termed malignant OHS is used to describe a severe multi-system disease due to the systemic effects of obesity. Patients with this syndrome have severe obesity-related hypoventilation together with systemic hypertension, diabetes, metabolic syndrome, left ventricular hypertrophy with diastolic dysfunction, pulmonary hypertension, and hepatic dysfunction [4]. Overweight and obesity increase all-cause mortality [5]. OHS as an independent risk factor for mortality in DKA patients has not been studied. Hyperglycemia causes an increase in mortality with an OR of >2.85 in patients with blood glucose > 300 mg/dl [6]. OHS is independently associated with increased mortality, and in the setting of another acute event like DKA, the odds of mortality, the requirement for mechanical ventilation, and acute kidney failure are higher. Our study also showed that the length of stay and cost of hospitalization are much higher in patients with coexistent DKA and OHS.

Outpatient treatment for OHS includes weight loss, bariatric surgery, continuous positive airway pressure (CPAP), and non-invasive ventilation (NIV) [7]. OHS is a relatively underdiagnosed medical condition and, if untreated, leads to increased morbidity and mortality and adverse health-related outcomes related to cardiovascular and respiratory complications [8-12]. Diabetes is an independent predictor of mortality in OHS [4]. This study emphasizes that the coexistence of diabetes and OHS could intensify the risk of mortality in acute conditions like DKA. Severe OHS should be treated as a systemic disease with respiratory, metabolic, and cardiovascular components that require a multi-model therapeutic approach [13]. Most OHS patients died from heart failure rather than respiratory failure and obesity did not have any effect on mortality [13].

OHS patients had higher mortality compared to simple obesity, it increases the burden of chronic inflammation, is a pro-inflammatory state, and work of breathing is higher in OHS compared to eucapnic obesity [14]. Diabetes is characterized by increased oxidative stress and endothelial dysfunction, which play a major role in diabetic vascular disease and the risk for complications related to the cardiovascular system [15]. OHS is also characterized by increased oxidative stress and decreased ventilatory drive, a theory correlated to hyperleptinemia, associated with a reduction in respiratory drive and reduced hypercapnia response irrespective of the amount of body fat. As leptin is a stimulant of ventilation, this study suggests an extension of leptin resistance to the respiratory center [16]. Central leptin resistance results in high plasma leptin and increased peripheral actions of leptin such as increased sympathetic outflow and cytokine production, which further contribute to adverse outcomes in DKA [17]. DKA is attributed to relative insulin insufficiency and excess production of stress hormones (glucagon, catecholamine, cortisol, and growth hormone) and results in metabolic acidosis [18]. Metabolic acidosis in DKA is compensated by an increase in the respiratory rate (Kussmaul breathing) and respiratory alkalosis, which is compromised in patients with OHS due to poor breathing, which could lead to adverse outcomes such as the need for invasive mechanical ventilation when they coexist and even respiratory failure. Hence, early recognition of OHS and initiation of NIV could be helpful and further research is necessary based on prospective studies to have a better understanding of the outcomes with the initiation of early vs. late NIV in patients hospitalized with coexisting OHS and DKA.

The study has some limitations. This is a retrospective cross-sectional study done using the NIS database, which cannot draw any conclusions on causal relationships but can strongly point toward any major associations. Data regarding adherence to medications in diabetic patients and compliance with CPAP vs. NIV in OHS are lacking. Duration of disease and lab parameters are not available in the database. Confounding bias can occur due to missing variables despite performing multivariate analysis for as many variables as possible. However, the generalizability of the database to the nation's population is a validated tool. The conclusion of this study is to recognize OHS as a possible comorbidity, and early initiation of NIV or CPAP might improve mortality and prevent mechanical ventilation in patients admitted with DKA.

## Conclusions

OHS is an independent risk factor for mortality in DKA, irrespective of the degree of obesity. Further prospective studies are recommended to study the effects of different treatment modalities of OHS such as the identification of the need for early NIV or for early invasive mechanical ventilation to improve outcomes in DKA patients.
